# IBD BioResource: an open-access platform of 25 000 patients to accelerate research in Crohn’s and Colitis

**DOI:** 10.1136/gutjnl-2019-318835

**Published:** 2019-07-03

**Authors:** Miles Parkes

**Keywords:** genetics, crohn’s’ disease, ulcerative colitis, biobank

Give us the tools and we will finish the job

-Winston Churchill

An alliance of clinicians, academics, research nurses, funders, coordinators, programmers and, most importantly, patients has come together in the UK to deliver a powerful new platform to accelerate Crohn’s and Colitis research—the inflammatory bowel disease (IBD) BioResource. As part of the National Institute for Health Research (NIHR) BioResource for translational research, 25 000 patients in over 90 hospitals UK-wide have signed up since we launched in January 2016 (figure 1). All have detailed phenotypes databased including Montreal classification,^1^ treatment response history (updated annually), surgical history and comorbidities (see IBD BioResource panel descriptive, Clinical data collection sheet and Health and Lifestyle questionnaire). Serum, plasma and DNA samples are banked; and genome-wide genetic profiling undertaken. Participants’ data and samples can be studied, and they themselves surveyed or recalled for resampling or downstream studies (see figure 2). Critically, such studies can be led by any UK or overseas investigator whether from the worlds of clinical research, pharmacovigilance, science or industry.

**Figure 1:**
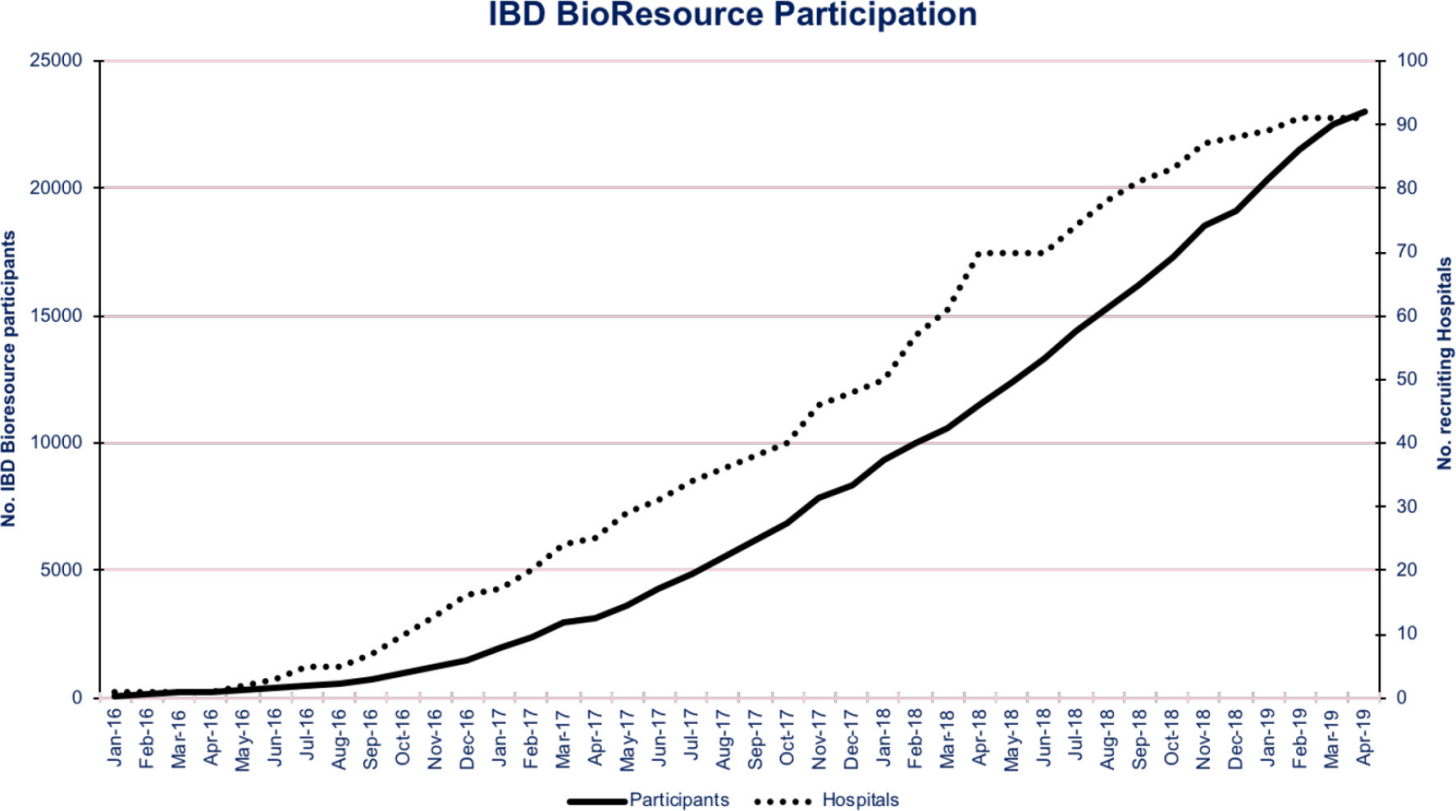
IBD BioResource recruitment in over 90 hospitals in the UK. IBD, inflammatory bowel disease.

**Figure 2:**
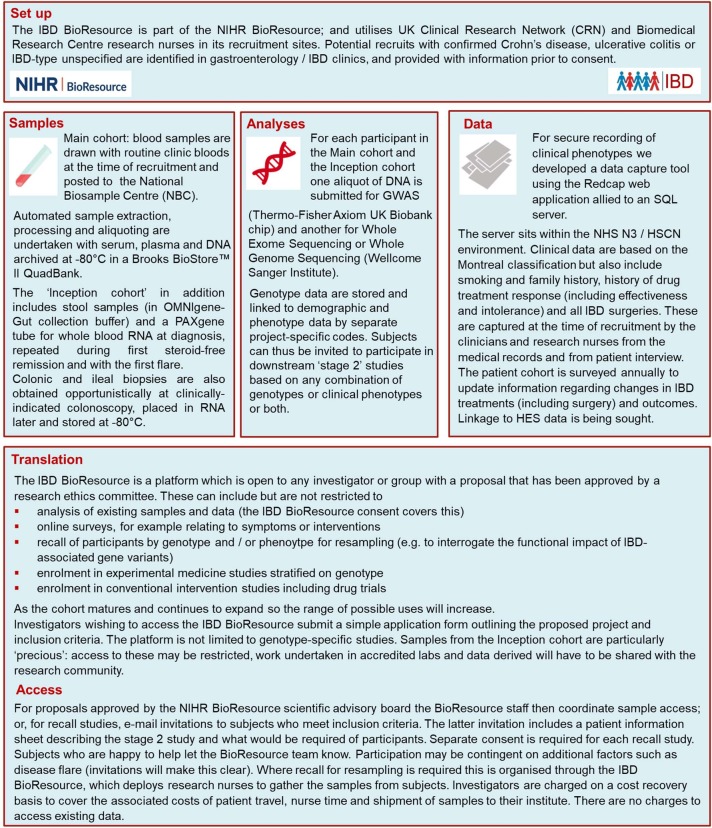
How the IBD BioResource works. HES, Hospital Episode Statistics; HSCN, Health Social Care Network; IBD, inflammatory bowel disease; NHS, National Health Service.

## What is the IBD BioResource for

A key motivation is to leverage recent genetics advances, and by understanding the functional impact of IBD-associated gene variants accelerate translation of the new knowledge for clinical benefit. Beyond this, it is increasingly evident that the IBD BioResource can facilitate a wide spectrum of research. This might include anything from mining existing data or samples or surveying the cohort regarding outcomes of newly licensed treatments, to pharmacogenetic research. It could also be used to expedite recruitment to intervention studies, including experimental medicine and conventional drug trials.

Gene discovery in Crohn’s disease and ulcerative colitis has placed these conditions at the forefront of the field of common disease genetics. As the UK IBD Genetics Consortium we have, through national and international collaboration, helped deliver a number of landmark studies in IBD.^2–14^ More than 240 confirmed IBD susceptibility loci have been identified to date. New druggable pathways continue to be identified and new pathogenic insights continue to accrue,^4^ particularly from those loci where the causal variants have been identified and functionally characterised. Examples of the latter include the association between IBD and the R381Q variant in the interleukin 23 receptor and between CD and the T300A variant in the autophagy gene ATG16L1.^15–17^

For most IBD risk loci, however, the causal genes and causal variants await identification. There are instances of hard-fought progress, with dissection of individual loci allowing new genes to be characterised and new biological insights gained^18 19^; and using innovative genetic ‘fine mapping’ techniques it has been possible to refine ~20% of risk loci to a single variant.^2^ However, functional characterisation of the latter is awaited, and is made all the more challenging by the fact that most are non-coding (regulatory). Furthermore, more than 50% map to genomic regions lacking homology with any known functional motif. The prize may be new biological insight not just about IBD pathogenesis but potentially also new understanding of the regulation of gene transcription, but no number of DNA samples can deliver this. Without recourse to mouse models or genetically manipulated cell lines, with all their problems, investigators will be able to use the IBD BioResource to access and resample cohorts of patients homozygous for risk and wild-type alleles at any locus. Already, despite only having recently reached maturity, such ‘stage 2’ recall-by-genotype studies are in progress. These are interrogating the functional impact of cytokine polymorphisms, non-coding RNA’s and HLA variants implicated in our genome wide association studies (GWAS), to better understand how these contribute to IBD pathogenesis and to specific subphenotypes.

Critical to the success of the IBD BioResource will be the willingness of patients to participate in annual surveys (to gather long term data re treatment outcomes etc) and stage 2 studies. It is early to be drawing conclusions, but in stage 2 studies to date 30%–60% of eligible participants have agreed to help. Maintaining engagement with patients and potential stage 2 users is clearly a key role of the team of four IBD BioResource coordinators and one data manager. As well as running the annual surveys, they maintain the website (www.ibdbioresource.nihr.ac.uk), circulate newsletters and promote activities through social media (Twitter and LinkedIn). Over the next 5 years, it is anticipated that longitudinal data from surveys will be substantially augmented by access to NHS digital and potential collaborations with the IBD Registry and Health Data Research UK, to minimise the risk of participants being lost to follow-up.

In addition to its recall/resample functionality, the IBD BioResource will also be used as the substrate for a new suite of genetics analyses. There is still more mileage in GWAS for risk variants: as the IBD dataset expands so the number of loci detected will increase in linear proportion, and new insights gained.^20^ Beyond GWAS increasing attention is turning to exome and whole genome sequencing, particularly to ascertain low frequency risk variants of potentially greater effect size than are typically identified by GWAS.^3^ These may contribute to the ‘missing heritability’ of IBD and other common diseases.^21^ Two limiting factors to date have been sequencing costs and the need for very large sample sizes to detect rare variant associations with statistical confidence. Both are becoming tractable. All IBD BioResource participants will undergo 50x exome sequencing or 20x genome sequencing and the data will be meta-analysed with other datasets in the international IBD genetics consortium in well powered studies; and the results made available to the research community.

## Translational aspects

As well as identifying susceptibility genes, there is increasing interest in identifying biomarkers for clinically relevant outcomes such as prognosis and treatment response, the aim being to deliver on the promise of personalised medicine. This requires detailed phenotypes on large sample sets—something the IBD BioResource has, with 95% completion of core data fields on the first 24 000 participants. Lee *et al* previously used GWAS to identify loci associated with Crohn’s disease prognosis^22^; and blood monocytes from genotype-selected NIHR BioResource subjects to interrogate the function of a FOXO3 polymorphism.^23^ The expanding IBD BioResource dataset should identify more markers associated with disease course and enable development of progressively more accurate ‘polygenic scores’ for prognosis. The latter use the power of genome-wide data (rather than just genome-wide significant hits), potentially combined with environmental modifyers such as smoking, to substantially improve predictive accuracy.^24^ This could be used at the time of IBD diagnosis to identify individuals destined for a complex disease course with multiple flares, perhaps warranting early and aggressive therapy.

Regarding treatment outcomes, pharmacogenetic analyses have recently identified a number of clinically important associations. Thus, NUDT15 polymorphisms are associated with thiopurine-induced leucopenia both in Asians and Europeans^25 26^; and human leukocyte antigen (HLA) DRB1*0701 is associated with risk of thiopurine pancreatitis.^27^ Other work lead by Tariq Ahmad in Exeter, including samples from the IBD BioResource, has identified strong association between HLA DQA1-05 (found in 40% of people of European origin) and immunogenicity to anti tumour necrosis factor (TNF) therapy.^28^ Studies to demonstrate the utility of testing for these in the IBD clinic is a near-term objective for IBD BioResource investigators.

Future pharmacogenetic studies will also benefit from the scale of the IBD BioResource. By recruiting from hospital clinics, it is biased towards more severely affected patients. Thus >13 000 have been treated with thiopurines and >9000 with anti-TNF therapy, all with detailed treatment outcomes recorded. Through the IBD BioResource, all participants can be recontacted or recalled if more detailed information or more samples are required. Even relatively recently introduced therapies are strongly represented, for example, over 1400 participants have received vedolizumab—and through an annual ‘treatment update’ survey, we expect to see this number climbing rapidly. We thus have a clear opportunity to better understand how these treatments are being used in routine practice, and for further pharmacogenetic analyses, for example, of treatment response. There may also be a role for the IBD BioResource in pharmacovigilance for new therapies.

## Supporting intervention studies and drug trials

Can the IBD BioResource directly support intervention studies, including drug trials? We believe so. Later this year, it will be used to recruit to the IBD BOOST study, surveying 12 000 patients and then recruiting 1180 with self-reported symptoms of fatigue, pain and/or urgency for different interventions. There is also potential utility for drug trials recruitment. There is widespread recognition that drug development takes too long, is too costly and potentially high risk.^29^ With the recent expansion of licensed IBD therapies industry may see the cost of developing a new drug, particularly one that ends up as fourth or fifth line therapy, as too great—and might shelve some of the potentially valuable new treatments.^30^ Different strategies are required, potentially including experimental and precision medicine to investigate subgroups of patients who have distinct pathogenic pathways and in whom drugs which target those pathways may demonstrate high efficacy (hence warranting earlier use in such biomarker-defined subsets). The IBD BioResource, with its detailed genome-wide data, is ideally positioned to support such studies.

At a more general level, the IBD BioResource is well placed to accelerate recruitment to conventional phase 3 studies—widely regarded as a major bottle-neck in drug development. This should be of great interest to the pharma industry and clinical research organisations. Potential participants meeting inclusion criteria for a particular study (identified from the IBD BioResource database of phenotypes and drug histories) and under a trial site hospital can be provided with trial details and notified that if their IBD is flaring and they are interested they should contact the research nurse at their site. Many patients are only too willing to participate in research but a major block is giving them the opportunity.^31^ Inherent to the problem is that most drug trial recruitment happens in clinic, but requires the clinician meeting the patient in flare to know about the study, be aware of inclusion criteria and to match these up to the patient in front of them. This presupposes an alignment of knowledge, time and motivation in busy clinicians, and all too often one or more of these is lacking. Having a large cohort of research-engaged IBD BioResource participants who have consented to screening of their medical records and can be contacted directly regarding research should help circumvent this bottleneck.

## The future

A key goal over the next 5 years is to recruit an inception cohort of 1000 individuals newly diagnosed with IBD who will undergo more detailed sampling (including stool, biopsy tissue and whole blood for RNA—unconfounded by the effects of drug treatment) and longitudinal follow-up. In due course, this will facilitate and expand existing research into the determinants, predictors and biomarkers of disease course and treatment response.^32 33^

The IBD BioResource has more than 90 hospitals participating UK-wide, and is now recruiting at ~1000 patients per month (figure 1). With 25 000 highly characterised patients already signed up, it is very definitely ‘open for business’ and now able to support and expedite a wide range of studies. What began as a resource to enable post-GWAS studies has become a wider multidimensional platform ready for use by the global IBD research community. Its job is to accelerate IBD research across all domains, improve treatments and outcomes, and perhaps one day ‘finish the job’ by achieving a cure. We are keen to see this tool used!

## To apply to use the IBD BioResource please visit

https://BioResource.nihr.ac.uk/researchers/researchers/application-process/

Enquiries re its use to ibd@bioresource.nihr.ac.uk
